# The impacts of climate change and disturbance on spatio‐temporal trajectories of biodiversity in a temperate forest landscape

**DOI:** 10.1111/1365-2664.12644

**Published:** 2016-03-28

**Authors:** Dominik Thom, Werner Rammer, Thomas Dirnböck, Jörg Müller, Johannes Kobler, Klaus Katzensteiner, Norbert Helm, Rupert Seidl

**Affiliations:** ^1^ Institute of Silviculture Department of Forest – and Soil Sciences University of Natural Resources and Life Sciences (BOKU) Vienna Peter‐Jordan Straße 82 1190 Vienna Austria; ^2^ Department for Ecosystem Research & Environmental Information Management Environment Agency Austria Spittelauer Lände 5 1090 Vienna Austria; ^3^ Bavarian Forest National Park Freyungerstraße 2 94481 Grafenau Germany; ^4^ Department of Ecology and Ecosystem Management Chair of Terrestrial Ecology Technische Universität München Hans‐Carl‐von‐Carlowitz‐Platz 2 85354 Freising Germany; ^5^ Institute of Forest Ecology Department of Forest‐ and Soil Sciences University of Natural Resources and Life Sciences (BOKU) Vienna Peter‐Jordan Straße 82 1190 Vienna Austria

**Keywords:** biodiversity hotspots, climate change impacts, conservation management, forest ecosystem management, insect diversity, Kalkalpen National Park, landscape ecology, plant diversity

## Abstract

1. The ongoing changes to climate challenge the conservation of forest biodiversity. Yet, in thermally limited systems, such as temperate forests, not all species groups might be affected negatively. Furthermore, simultaneous changes in the disturbance regime have the potential to mitigate climate‐related impacts on forest species. Here, we (i) investigated the potential long‐term effect of climate change on biodiversity in a mountain forest landscape, (ii) assessed the effects of different disturbance frequencies, severities and sizes and (iii) identified biodiversity hotspots at the landscape scale to facilitate conservation management.
2. We employed the model iLand to dynamically simulate the tree vegetation on 13 865 ha of the Kalkalpen National Park in Austria over 1000 years, and investigated 36 unique combinations of different disturbance and climate scenarios. We used simulated changes in tree cover and composition as well as projected temperature and precipitation to predict changes in the diversity of Araneae, Carabidae, ground vegetation, Hemiptera, Hymenoptera, Mollusca, saproxylic beetles, Symphyta and Syrphidae, using empirical response functions.
3. Our findings revealed widely varying responses of biodiversity indicators to climate change. Five indicators showed overall negative effects, with Carabidae, saproxylic beetles and tree species diversity projected to decrease by more than 33%. Six indicators responded positively to climate change, with Hymenoptera, Mollusca and Syrphidae diversity projected to increase more than twofold.
4. Disturbances were generally beneficial for the studied indicators of biodiversity. Our results indicated that increasing disturbance frequency and severity have a positive effect on biodiversity, while increasing disturbance size has a moderately negative effect. Spatial hotspots of biodiversity were currently found in low‐ to mid‐elevation areas of the mountainous study landscape, but shifted to higher‐elevation zones under changing climate conditions.
5. *Synthesis and applications*. Our results highlight that intensifying disturbance regimes may alleviate some of the impacts of climate change on forest biodiversity. However, the projected shift in biodiversity hotspots is a challenge for static conservation areas. In this regard, overlapping hotspots under current and expected future conditions highlight priority areas for robust conservation management.

## Abstract 2

Our results highlight that intensifying disturbance regimes may alleviate some of the impacts of climate change on forest biodiversity. However, the projected shift in biodiversity hotspots is a challenge for static conservation areas. In this regard, overlapping hotspots under current and expected future conditions highlight priority areas for robust conservation management.

## Introduction

Biodiversity has been identified as a key determinant for the quality and functioning of ecosystems world‐wide (Díaz & Cabido 2001; Cardinale, Palmer & Collins 2002). The currently ongoing decline in biodiversity threatens the ability of ecosystems to adapt to changing conditions and hampers the provisioning of ecosystem services, and thus represents one of the greatest challenges for humanity (Bellard *et al*. 2012). Changes in land use and climate have been identified as the main drivers of this decline (Sala 2000). Particularly, forest ecosystems are under pressure, as climate change may threaten forest‐dependent species across a wide range of species groups (Thomas *et al*. 2004). The vulnerability of forest biodiversity along with the fact that the majority of terrestrial species depend on forest ecosystems underlines the key role of forests in conservation management (Myers *et al*. 2000; Parrotta, Wildburger & Mansourian 2012).

The majority of studies on climate change impacts on biodiversity have focused on direct effects of climate change, that is effects of changes in temperature and precipitation on biodiversity. Fewer works have also investigated indirect effects, such as the effect of climate‐mediated changes in forest structure and composition on species presence and abundance (e.g. De Frenne *et al*. 2013). While forest structure and composition generally respond slowly to environmental changes, they can be altered quickly and profoundly by disturbances, that is pulses of tree mortality caused by agents such as bark beetles, fire and wind. Disturbances are climate sensitive and have already intensified during the last decades (Seidl, Schelhaas & Lexer 2011). A further intensification of disturbance regimes in response to ongoing climatic changes is likely (Seidl *et al*. 2014; Millar & Stephenson 2015). While often regarded as undesirable ‘calamities’ in forest management, the resulting increases in biodiversity (e.g. indicated by the number of species) generally reveal a positive impact of disturbances on biodiversity (Müller *et al*. 2008; Thom & Seidl 2015). However, the net effect of changing climate and disturbance regimes on forest biodiversity remains unclear: will intensifying disturbance regimes offset the predicted negative direct effects of climate change on biodiversity? Or will increasing climate and disturbance change threaten the ecological resilience of ecosystems and consequently the habitat quality of forest‐dependent species?

Future climate change impacts on plant and animal diversity have predominantly been assessed using niche models, that is empirical relationships between species presence or abundance and climate variables (Zimmermann *et al*. 2010). Notwithstanding their scientific value (e.g. assessing the climatic suitability of species and their potential range for migration under future climate), such models have major shortcomings in the context of conservation planning and management. For instance, they commonly ignore biotic interactions that strongly affect species composition [but see Thuiller *et al*. (2015)]. Moreover, niche models assume that species track changing climatic conditions instantaneously, disregarding time‐lags and indirect effects of climate change such as habitat changes and disturbance‐driven perturbations (Elith & Leathwick 2009). In contrast, process‐based forest simulation models project transient pathways of ecosystem change while accounting for the complex and interacting effects of climate change (Kearney & Porter 2009). These approaches, however, usually focus solely on tree vegetation and rarely address other species relevant in the context of biodiversity conservation.

Here, we combined landscape‐scale forest simulation modelling with empirical climate–diversity relationships to circumvent many of these limitations. Our aim was to address the climate sensitivity of forest biodiversity explicitly in space and time, and particularly study the effect of current and changed climate and disturbance regimes over an extended time frame of 1000 years. Our specific objectives were to (i) investigate the role of climate change on a wide range of indicators of forest biodiversity over time, (ii) assess the effects of different disturbance frequencies, severities and sizes on biodiversity indicators and (iii) identify current and future biodiversity hotspots at the landscape scale to facilitate future conservation management. The latter question is of particular relevance as identifying and preserving areas of particular value for biodiversity, for example due to their particular richness or habitat value for keystone species (Myers *et al*. 2000), is a cornerstone of current conservation management. Many existing protected areas are centred on such biodiversity hotspots, yet whether these systems also will remain hotspots in a drastically changing climate remains uncertain (see e.g. Hansen *et al*. 2001; Bässler *et al*. 2013). Here, we tested for a shift of biodiversity hotspots along the steep altitudinal gradients of our study landscape to higher‐elevation areas due to reduced thermal limitations in a future climate. Based on previous large‐scale assessments, we furthermore hypothesized an overall negative impact of climate change on forest biodiversity (Thomas *et al*. 2004), but a positive effect of natural disturbance (Thom & Seidl 2015). Finally, we tested the hypothesis that a slow response of forest composition and structure leads to a considerable time‐lag in the response of biodiversity to changing climatic conditions (Bertrand *et al*. 2011).

## Materials and methods

### Study area

**Figure 1:**
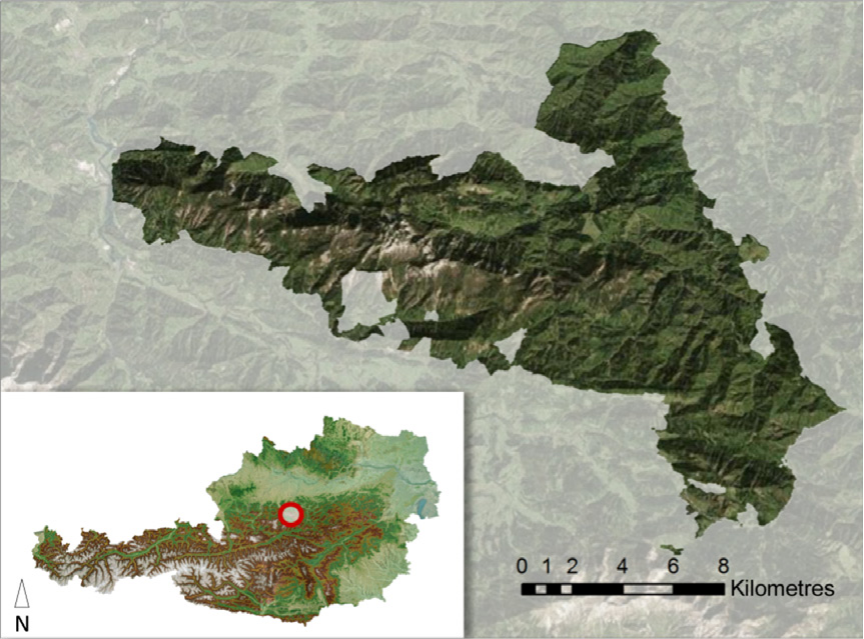
Location, extent and topography of the study landscape – Kalkalpen National Park. [Colour figure can be viewed at wileyonlinelibrary.com].

The Kalkalpen National Park (KA‐NP) is located at N47.47° E14.22°, in the northern front range of the Austrian Alps (Fig. 1). The landscape is characterized by steep mountainous terrain, with elevations ranging from 385 to 1963 m a.s.l. Soils are predominately shallow with Lithic and Rendzic Leptosols and Chromic Cambisols as the dominant soil types over calcareous bedrock. The climate varies with topography, with temperature decreasing (mean annual temperature range: 3·6–9·0 °C) and precipitation increasing (mean annual precipitation range 1205–1741 mm) with elevation. With a total size of 20 856 ha mainly consisting of forests, the Kalkalpen National Park is the largest forest wilderness in Austria. It includes a diverse range of forest ecosystems including European beech *Fagus sylvatica* (L.) forests in the lower reaches, mixed forest types of beech, Norway spruce *Picea abies* (L. Karst.) and silver fir *Abies alba* (Mill.) in mid‐elevations and subalpine spruce forests in high elevations. Before establishment of the KA‐NP in 1997, the area was managed mainly for timber production, but today conforms to IUCN category II (National Park).

### Simulation model

To simulate forest landscape dynamics at KA‐NP, we used iLand, the individual‐based forest landscape and disturbance model. iLand is a spatially explicit process‐based model. It was developed to simulate interactions between environmental drivers (e.g. climate regime, nutrient and water availability), forest vegetation processes (e.g. growth, mortality and regeneration) and disturbances regimes (e.g. windstorms, wildfires) (Seidl *et al*. 2012a). Processes in iLand interact in a hierarchical multiscale framework, including processes on the tree (e.g. growth, mortality, competition for resources), stand (availability of water, nutrients) and landscape level (disturbance, seed dispersal). The simulation of primary production in iLand is based on a light‐use efficiency approach, with scalar modifiers accounting for the effects of temperature, soil water availability, vapour pressure deficit, nutrient availability as well as atmospheric carbon dioxide (CO_2_) concentration. Mortality of trees in iLand considers intrinsic mortality (i.e. age‐related causes) and stress‐related mortality (using carbon starvation as a physiological proxy for stress) as well as disturbance events such as windstorm or fire. Regeneration is spatially explicit and depends on the availability of seeds as well as favourable light and environmental conditions. A detailed description of iLand is available in Seidl *et al*. (2012a,b) and from an extensive online documentation (http://iLand.boku.ac.at).

For the current study, we evaluated the model's ability to simulate the KA‐NP by testing iLand's efficiency to reproduce expected values of productivity (Fig. S1, Supporting information), climate sensitivity (Fig. S2) and the potential natural vegetation (Fig. S3). Overall, these tests resulted in good correspondence of the model with independent observations and supported the application of iLand for studying ecosystem dynamics at the KA‐NP.

### Initial conditions and drivers

Soil and climate varied at a spatial grain of 100 × 100 m in the simulations (in total 19 200 ha), while the initial vegetation conditions were derived for stand polygons covering the 13 865 ha forest area of the KA‐NP (median stand size: 1·4 ha). Soil depth and type (Kobler 2004), texture (from inventory plots) as well as plant available nitrogen (Seidl, Rammer & Lexer 2009) were used to characterize soil conditions. To initialize the current vegetation, we combined data sources from forest inventory and planning data, aerial photo analysis and LiDAR. Altogether, we initialized more than 2 10^6^ trees from 17 different species, representing the state of the tree vegetation at KA‐NP in the year 1999. Four climate scenarios were studied: a baseline climate scenario where we repeatedly sampled years from the period 1950–2010 for the 1000‐year simulation period, and three regionally downscaled climate change scenarios, representing different combinations of global and regional circulation models under A1B forcing. A stabilization of climate conditions at the level of 2080–2099 was assumed for the years beyond 2100 (i.e. *inter alia* 3·1–3·3 °C change relative to the baseline period). A more detailed description of the initial conditions and driver data for the simulation is provided in Appendix S1.

### Landscape simulation

We simulated the currently forested 13 865 ha of the KA‐NP for 1000 years with 36 unique combinations of climate and disturbance scenarios to derive tree species composition at the level of 100‐m grid cells. In addition to the four climate scenarios described above, we investigated all possible combinations of two different disturbance frequencies, severities and size scenarios for every climate scenario. The low‐intensity disturbance variant represents the current disturbance regime, with a rotation period of 250 years (Thom *et al*. 2013) and a mean disturbance size of 5·3 ha (based on a disturbance inventory at KA‐NP). As moderate disturbance severity, we assumed a mortality of 50% of trees with diameter at breast height (d.b.h.) > 10 cm in this variant. At increased disturbance scenarios, the disturbance rotation period was halved to 125 years, the size increased 10‐fold to 53·4 ha, and the severity doubled to 100%. Additionally, we included a scenario without disturbance, bringing the total number of studied disturbance scenarios to nine (2 frequencies × 2 severities × 2 sizes + 1 no disturbance scenario). Within these disturbance regime definitions, simulated disturbances were implemented stochastically in each scenario, with the actual disturbance size drawn from a negative exponential distribution, and the location of the disturbance assigned randomly to the landscape. Each scenario was replicated ten times to account for stochasticity in the simulation. We tested the sufficiency of using ten replicates per scenario by analysing the coefficients of variation (cv) of our response variables, and found robust results for all indicators at the end of the simulation period (cv < 2% for all indicators and scenarios, Table S1). In total, 360 simulations were conducted (four climate scenarios × nine disturbance scenarios × 10 replicates). This simulation design was specifically developed (i) to stringently distinguish climate and disturbance effects on biodiversity (due to independence of climate and disturbance scenarios in the simulation) and (ii) to assess which disturbance regime parameters (i.e. size, severity or frequency) are most influential on biodiversity. To account for the vegetation changes that have occurred between 1999 (the year for which initial vegetation information was available) and 2013 (the initial year of the analysis), we ran the model for these 14 years using the respective climate forcing and recreating the disturbances that were observed during that period. Each simulation was then run over 1000 years starting in the year 2013.

### Indicators of biodiversity

To obtain a comprehensive assessment of the climate and disturbance effects on forest biodiversity, we jointly analysed eleven different biodiversity indicators for each simulated 1‐ha grid cell. Tree species diversity and canopy complexity were directly derived from iLand simulations. Basal area shares were used to compute tree species diversity, using the exponent of the Shannon index [exp(*H'*)] as an indicator for the effective number of tree species. Canopy complexity was described by means of the rumple index (Parker *et al*. 2004), which is the ratio of the canopy surface area to the projected ground surface area, calculated here at 10‐m horizontal resolution. With regard to animal diversity, richness data (the number of species) on Araneae (web spiders), Carabidae (ground beetles), Hemiptera (true bugs), Hymenoptera (sawflies, wasps, bees and ants), Mollusca (snails and slugs), saproxylic (deadwood‐dependent) beetles, Symphyta (sawflies), as well as Syrphidae (hoverflies), were derived from biodiversity inventories (0·1‐ha plots) in 52 locations distributed over neighbouring Bavaria (Bässler *et al*. 2008). Furthermore, data on the richness of the ground vegetation (vascular plant species with a height of up to 60 cm) were derived from the FlorAlp data base (Dullinger *et al*. 2012) by selecting releves with a uniform size of 625 m^2^ (*n* = 852). Based on these data, we developed empirical response functions for the nine biodiversity indicators not derived directly from simulations, where the response variable (the number of species in each group) was related to mean annual precipitation sum (*P*_sum_) and mean annual temperature (Tmean) (indicators of the climate regime), canopy cover (an indicator for light availability and the local thermal regime) and the relative share of canopy tree species (indicators of species association). We used negative binomial generalized linear models (glms) with a logarithmic link function to predict species diversity of each indicator. Based on ecological theory, we hypothesized an optimum relationship of temperature and canopy cover for each indicator and consequently transformed these predictors using second‐order polynomial functions (Austin 2002). The transformed variable was retained if the species diversity response was biologically meaningful. To determine the model most strongly supported by the data, we used Akaike's Information Criterion (AIC), Nagelkerke *R*² values as well as *P*‐values from chi‐square goodness‐of‐fit tests. Final models were tested for multicollinearity by means of (generalized) variance inflation factors (VIF or GVIF). To further analyse the thus‐derived empirical models with regard to their response to climate and tree vegetation changes, a local sensitivity analysis was conducted.

In a subsequent step, we used the fitted glms with the respective climate input and iLand‐derived tree layer information to project biodiversity responses for all scenarios. For each of the eleven indicators, we derived the effect of climate change at any given point in space and time by relating each simulation under climate change to the mean over the baseline period under the same disturbance scenario. For the analyses of climate change effects over time, we aggregated the 100‐m grid cells to landscape‐level mean responses. From these comparisons over all scenarios and replicates, the median and the 95th percentile range of climate‐induced diversity changes were computed. Similarly, the disturbance effect was calculated by relating scenarios of different disturbance frequency, severity and size to the respective undisturbed scenario under any given climate regime. Both climate and disturbance effects were tested against the null hypothesis of no effect by means of Wilcoxon's signed rank sum test.

### Biodiversity hotspots

To also address spatial changes in biodiversity on the landscape, we assessed biodiversity hotspots at KA‐NP; to that end, we identified areas that support a high diversity across all indicators. To be able to compare across indicators, we used percentiles of diversity estimates for all 360 simulations at the end of the simulation period. In analogy to the assessment of multifunctionality across ecosystem services (e.g. Pasari *et al*. 2013), hotspots were defined as area where every indicator reaches or exceeds a predefined threshold (here set this at the 25th percentile value). Differences between scenarios were analysed by means of McNemar's chi‐squared test, and spatial analysis of hotspots was conducted by mapping at a grain of 100‐m grid cells. To evaluate sensitivities of the result to this particular definition of hotspots, an alternative hotspot definition was also investigated (see Fig. S4 for details).

## Results

### Sensitivity of forest biodiversity

The empirical models for predicting diversity in species groups were found to satisfactorily fit the empirical data, with pseudo‐*R*² values ranging from 0·23 to 0·96 (Table 1). Chi‐square goodness‐of‐fit tests did not reject the final models. VIF and GVIF, respectively, were all <10, indicating that final models were not strongly affected by multicollinearity (Dormann *et al*. 2013).

**Table 1:** Parameters and goodness‐of‐fit of the empirical species diversity models (negative binomial generalized linear models with a logarithmic link function). *T*_mean_: mean annual temperature; *P*_sum_: sum of annual precipitation; beech, spruce, oak + hornbeam as well as canopy cover are relative shares (%). poly() indicates the polynomial transformation (second order) of a predictor

Response variable	Predictors	*R*² (Nagelkerke)	*P*‐value (Chi²)
Araneae	*T*_mean_, *P*_sum_, oak + hornbeam, poly(canopy cover)	0·61	0·179
Carabidae	*T*_mean_, *P*_sum_, beech, canopy cover	0·26	0·126
Ground vegetation	poly(*T*_mean_), *P*_sum_, spruce, poly(canopy cover)	0·23	0·176
Hemiptera	*T*_mean_, beech, spruce, canopy cover	0·54	0·252
Hymenoptera	*T*_mean_, *P*_sum_, poly(canopy cover)	0·90	0·174
Mollusca	*T*_mean_, spruce, canopy cover	0·87	0·250
Saproxylic beetles	poly(*T*_mean_), *P*_sum_, oak + hornbeam, poly(canopy cover)	0·96	0·173
Symphyta	*T*_mean_, beech, poly(canopy cover)	0·37	0·108
Syrphidae	*T*_mean_, *P*_sum_, beech, poly(canopy cover)	0·47	0·186

Analyses of the fitted parameters and sensitivity analysis indicated that taxonomic groups reacted non‐uniformly to modifications in their environment (Table S2). Changes in average temperature, for instance, resulted in a range of responses: while species groups such as Hymenoptera and Syrphidae strongly benefited from increasing temperatures (+71·8% and +49·9% for a + 1 °C increase), others such as Araneae and saproxylic beetles were clearly negatively affected (−9·3% and −8·8% for the same temperature increase). Compared to this distinct temperature effect, precipitation had a weaker influence on diversity in species groups. Six out of nine models maintained precipitation as covariate though, with Araneae, Carabidae, Hymenoptera and saproxylic beetles showing negative responses, while ground vegetation and Syrphidae responding positively to an increase in precipitation. Besides the impacts of changing climatic conditions, changes in tree vegetation were also important determinants of diversity in species groups. While the proportion of beech and spruce was found to have negative impacts on biodiversity, the share of oak *Quercus petraea* (Matt.) and *Quercus robur* (L.) and hornbeam *Carpinus betulus* (L.) positively influenced diversity in a range of species groups. An increase in canopy cover was found to have negative effects on the species diversity of most taxonomic groups (between −1·8% and −13·6% for a 10% increase in canopy cover) – only Carabidae and saproxylic beetles were weakly positively related to canopy cover.

### Changes in biodiversity in response to climate change

**Figure 2:**
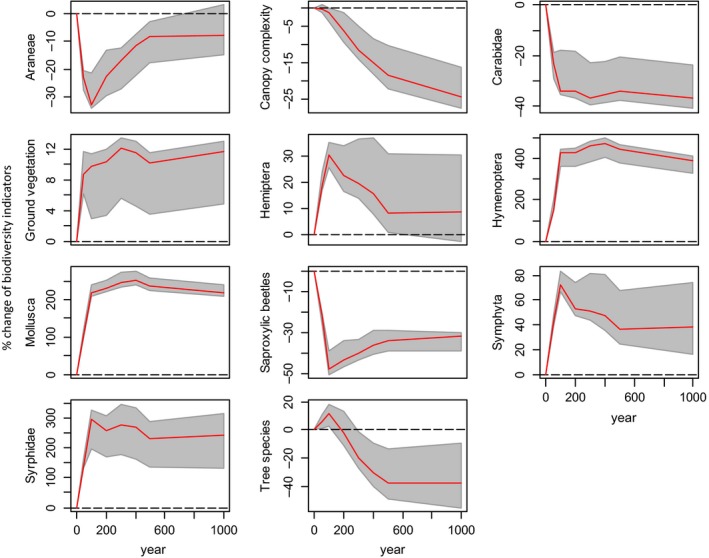
Landscape‐scale response of biodiversity indicators to climate change relative to baseline climate conditions (1950–2010). Red lines present the median, and grey shaded areas illustrate the 95th percentile range. The *y*‐axis indicates the percentage change compared to baseline climate conditions, while the *x*‐axis indicates the simulated year. Note that y‐axes are on different scales. [Colour figure can be viewed at wileyonlinelibrary.com]

Our simulations indicated a pronounced increase in the share of European beech and a decrease in Norway spruce under climate change (Table 2), with changes progressing considerably beyond the assumed point of climate stabilization in 2100. Tree species composition did, however, also change under baseline climate conditions, highlighting past management legacies in the current tree species composition. The combined effects of direct and indirect responses to climate change on the eleven biodiversity indicators were strongly divergent. While six indicators showed overall positive responses, five were negatively affected at the end of the 1000‐year simulation period (*P *<* *0·001). Climate change was beneficial for the diversity of ground vegetation, Hemiptera, Hymenoptera, Mollusca, Symphyta and Syrphidae, but reduced the diversity of Araneae, Carabidae and saproxylic beetles as well as the canopy complexity of forests in the landscape (Fig. 2, Table 3). Tree species diversity was slightly positively affected during the first 100–200 years, but eventually dropped to −35·8% compared to baseline climate conditions. The most drastic changes in a wide range of species groups were found during the first 100 years of the simulation, indicating a prominent direct climate effect. Tree layer‐mediated indirect effects were most distinctive for Araneae and saproxylic beetles, where the increase in oak and hornbeam cover in response to warming (positive indirect effect) compensated direct negative impacts of elevated temperature and reduced precipitation over the long term. The opposite signal was found for Hemiptera and Symphyta: while direct climate change effects were beneficial for both species groups, the climate‐induced increase in beech negatively influenced species diversity in these groups.

**Table 2:** The sensitivity of forest composition to climate change and disturbance. Values are based on iLand simulations and indicate means and standard deviations (SD) over averaged landscape values (i.e. average species shares in the landscape) of all respective scenarios

	Disturbance	Initial state	Baseline climate				Climate change			
		Year 0	Year 100		Year 1000		Year 100		Year 1000	
		Mean	Mean	SD	Mean	SD	Mean	SD	Mean	SD
*A. alba* (%)	No	–	3·3	0·0	13·1	0·0	3·6	0·1	1·3	1·0
	Yes	2·9	3·4	0·1	7·8	1·8	3·6	0·2	0·9	0·8
*C. betulus* (%)	No	–	<0·1	0·0	0·0	0·0	0·2	0·1	0·7	0·2
	Yes	<0·1	<0·1	0·0	0·1	0·1	0·4	0·3	2·8	1·5
*F. sylvatica* (%)	No	–	34·7	0·0	34·4	0·0	37·9	1·2	67·3	9·0
	Yes	39·3	27·7	4·0	22·2	4·1	31·6	3·8	49·7	11·5
*L. decidua* (%)	No	–	12·0	0·1	7·9	0·0	12·4	0·9	2·5	0·6
	Yes	10·7	12·5	0·3	8·4	0·8	13·0	1·0	4·6	1·2
*P. abies* (%)	No	–	43·2	0·1	21·5	0·1	36·8	0·7	2·2	0·2
	Yes	38·5	39·8	3·0	18·2	2·2	32·7	3·1	2·7	0·2
*Q. petraea* (%)	No	–	0·1	0·0	0·9	0·0	0·4	0·2	16·3	7·1
	Yes	<0·1	0·1	0·0	0·7	0·1	0·6	0·3	15·8	5·9
*Q. robur* (%)	No	–	0·1	0·0	0·5	0·0	0·3	0·1	4·6	1·3
	Yes	<0·1	0·1	0·0	0·5	0·0	0·4	0·2	6·6	1·9
Other tree species (%)	No	–	6·7	0·0	21·8	0·0	8·6	0·3	5·2	0·5
	Yes	8·6	16·3	7·0	42·1	8·7	17·7	6·2	16·9	6·6

**Table 3:** Response of biodiversity indicators to disturbance and climate scenarios for the years 0, 100 and 1000 of the simulation. Mean and standard deviation (SD) are over averaged landscape values (i.e. average species number in the landscape) for the respective scenarios. Presented are richness levels for Araneae, Carabidae, Hemiptera, Hymenoptera, Mollusca, saproxylic beetles, Symphyta and Syrphidae, the effective tree species diversity [exp(*H'*)] as well as the rumple index of forest canopy complexity

	Disturbance	Initial state	Baseline climate				Climate change			
		Year 0	Year 100		Year 1000		Year 100		Year 1000	
		Mean	Mean	SD	Mean	SD	Mean	SD	Mean	SD
Araneae	No	–	11·6	0·0	11·8	0·0	8·1	0·7	10·7	0·7
	Yes	10·7	11·6	0·0	11·8	0·0	8·2	0·6	11·2	0·7
Canopy complexity	No	–	1·2	0·0	1·5	0·0	1·2	0·0	1·1	0·0
	Yes	1·2	1·3	0·0	1·6	0·1	1·3	0·0	1·3	0·0
Carabidae	No	–	8·8	0·0	9·0	0·0	6·2	0·7	5·8	0·6
	Yes	7·4	8·9	0·1	9·2	0·0	6·3	0·7	6·1	0·6
Ground vegetation	No	–	33·1	0·0	34·0	0·0	35·6	1·1	37·3	1·2
	Yes	37·4	33·4	0·2	34·2	0·2	36·1	1·1	37·6	1·2
Hemiptera	No	–	33·4	0·0	39·5	0·0	43·7	0·9	43·9	4·9
	Yes	37·8	37·3	2·6	46·1	2·8	48·8	3·5	52·3	6·7
Hymenoptera	No	–	25·6	0·0	26·3	0·0	129·4	8·2	123·0	7·7
	Yes	37·5	26·0	0·3	26·7	0·3	132·7	8·6	127·2	8·4
Mollusca	No	–	10·1	0·0	11·9	0·0	32·7	1·2	38·9	1·3
	Yes	12·0	10·3	0·2	12·1	0·1	33·5	1·3	38·9	1·3
Saproxylic beetles	No	–	48·0	0·0	49·9	0·0	25·9	2·4	32·1	1·2
	Yes	44·5	48·1	0·1	49·8	0·0	26·0	2·3	33·5	1·2
Symphyta	No	–	11·4	0·0	10·9	0·0	19·7	0·5	14·7	2·0
	Yes	14·8	12·2	0·5	12·3	0·6	21·3	1·1	17·8	2·6
Syrphidae	No	–	19·9	0·0	18·9	0·0	73·6	10·2	59·8	12·4
	Yes	30·7	21·0	0·6	20·7	0·7	78·3	11·3	68·9	14·1
Tree diversity	No	–	3·1	0·0	5·7	0·0	3·4	0·1	2·8	0·3
	Yes	2·5	4·1	0·6	7·4	0·5	4·6	0·7	5·0	1·2

### Disturbance effects on biodiversity

**Figure 3:**
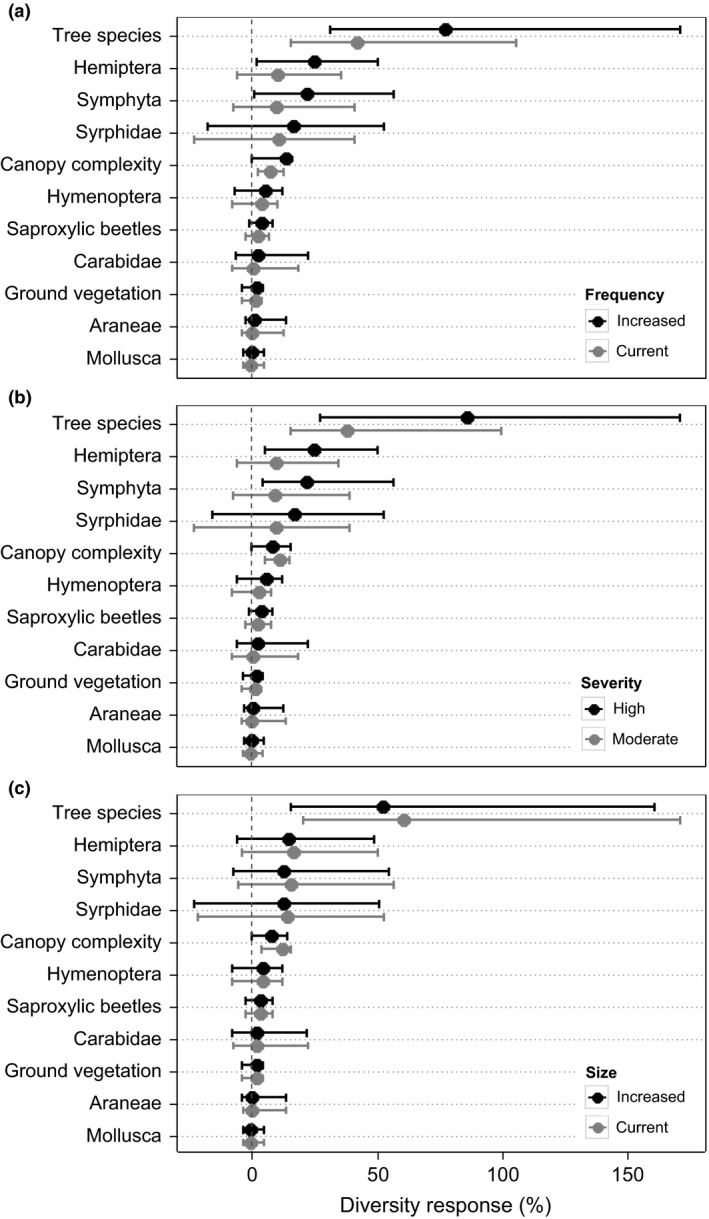
Response of biodiversity indicators to disturbance (a) frequency, (b) severity and (c) size relative to scenarios without disturbance (zero line) at the end of the 1000‐year simulation period. Dots are median values and whiskers indicate the 95th percentile range across all scenarios. Positive values indicate an increase in diversity.

While climate change impacts on diversity were ambiguous, but strong for each indicator studied, the effect of disturbance was generally positive, but less pronounced (Table 3, Fig. 3). Compared to a hypothetical trajectory omitting disturbances for the entire 1000‐year simulation period, disturbances increased diversity in all indicators (*P *<* *0·001). Tree species diversity was affected most strongly by disturbance, followed by the species groups Symphyta, Hemiptera, Syrphidae as well as canopy structure. Increases in both disturbance frequency and severity were positively associated with all biodiversity indicators (Fig. 3). The opposite was the case for disturbance size, where an increase in the mean disturbance size was found to decrease biodiversity.

### Shifts in biodiversity hotspots

**Figure 4:**
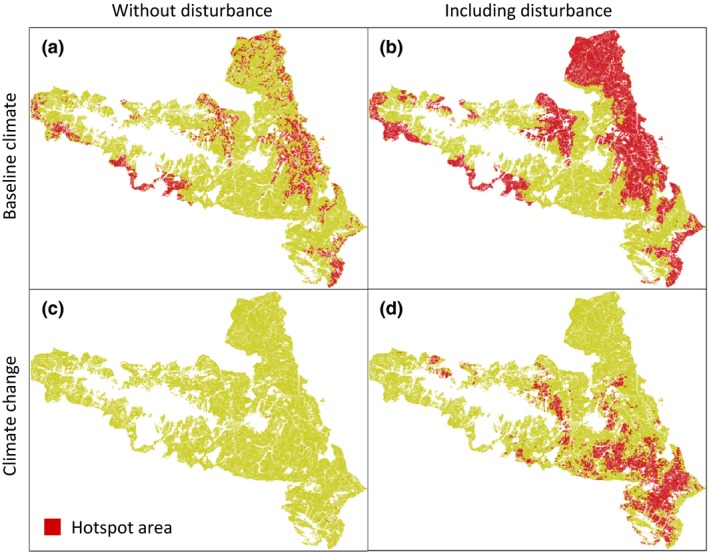
Biodiversity hotspots of the Kalkalpen National Park after 1000 simulation years. Hotspots are defined as areas where each of the eleven biodiversity indicators studied here exceeds the 25th percentile of its value range. [Colour figure can be viewed at wileyonlinelibrary.com]

After 1000 years of simulation, areas identified as hotspots were substantially different when comparing baseline climate and climate change conditions (Fig. 4). While hotspots in the baseline climate scenario were mainly located at low to moderate elevation, climate change supported hotspots in higher‐elevation zones. The extent of hotspot area strongly decreased under changed climatic conditions (*P *<* *0·001). Without disturbance and climate change, 17·2% of the landscape were hotspots (Fig. 4a), but only a heavily fragmented 0·1% remained under changed climatic conditions (Fig. 4c). Disturbance significantly increased the extent of hotspot areas (*P *<* *0·001), for example by 146·1% under baseline climate (Fig. 4b). Under future climate, the simulations resulted in a total hotspot area of at least 18·4% (Fig. 4d), of which 23·1% overlapped with current hotspots.

## Discussion

### Consequences of changes in climate and disturbance regimes

Based on the findings of broad‐scale assessments (e.g. Sala 2000; Thomas *et al*. 2004), we had hypothesized a decline in biodiversity in response to climatic changes for the KA‐NP. However, our in‐depth analyses revealed that climate change effects on forest biodiversity in mountain forest landscapes can be both positive and negative, depending on the indicator and species group assessed. This finding corroborates other studies showing divergent effects among taxonomic groups and exemplifies the existence of a considerable variation in local‐scale biodiversity trends (see e.g. Bowler *et al*. 2015). The steep altitudinal gradient of the study landscape (>1000 m) allows trees to migrate upwards, tracking their suitable climate niche. Doing so, European beech increasingly occupies areas dominated by Norway spruce, while giving way to oak and hornbeam at lower elevations. From this setting emanates a specific climate change response in biodiversity, which does not necessarily mirror broad‐scale biodiversity trends, as species linked to overstorey tree species [e.g. saproxylic beetles associated with oak (Bergman *et al*. 2012)] mainly track the spatio‐temporal shifts of these trees.

Another reason for the differentiated result of climate change impacts could be that many previous broad‐scale studies have considered the direct effects of climate change only. While individual studies have already incorporated selected indirect climate change effects previously, such as modifications in seed dispersal and biotic interactions (e.g. Brooker *et al*. 2007) or changes in forest structure (e.g. De Frenne *et al*. 2013), here we applied a novel combination of simulation modelling and empirical climate–diversity relationships to comprehensively assess both direct and indirect impacts of climate change. While all species groups indicated a strong direct response to climate, indirect effects also had a distinctive effect on the trajectories of a number of species groups studied (e.g. Araneae, saproxylic beetles, see Fig. S4). In line with our initial hypothesis, these indirect effects of climate change were considerably delayed; that is, forest structure and composition reacted slowly to changes in climatic conditions, resulting in a delayed response of other species groups. Also direct climate change impacts could be delayed, an effect that was not accounted for here. As the species groups investigated here have relatively short life cycles compared to the pace of climate change, a swift direct response to a changing environment can be assumed (see e.g. Danks 2004), and the lag of direct climate effects might be negligible in our study. Furthermore, our study does not consider biotic interactions within the investigated species groups or any associations with other species groups except trees (see e.g. Thuiller *et al*. 2015).

It is also important to note that the different definitions and focal indicators of biodiversity under consideration in different studies likely account for diverging reports on the climate change sensitivity of biodiversity. For ten out of eleven indicators, we here used species diversity as a proxy for biodiversity. Other important aspects of biodiversity not considered here include the abundance of rare species (e.g. red list species) or endemic species (Engler, Guisan & Rechsteiner 2004), as well as the consideration of functional diversity (Thuiller *et al*. 2006) or phylogenetic diversity (Thuiller *et al*. 2011). Moreover, despite the fact that we studied eleven different biodiversity indicators spanning the plant and animal kingdoms, it would be desirable to also include, for instance, vertebrate species such as mammals, birds, amphibians or reptiles (see e.g. Maiorano *et al*. 2013) in future assessments.

In contrast to climate change, disturbance had a clear positive effect on the investigated forest biodiversity indicators, supporting our initial hypothesis. This result is in line with a wide range of literature on the impacts of disturbance on biodiversity in forest ecosystems (Thom & Seidl 2015). However, we found different biodiversity responses for changes in the frequency, severity and size of disturbance. While the effect of an increase in disturbance frequency and severity was consistently positive, an increase in disturbance size reduced the positive disturbance effect on biodiversity. High disturbance severity and increasing frequency facilitate edges, and create a complex pattern of open areas and remaining closed canopy forests, increasing the variation in ecological conditions and habitats (Perry *et al*. 2011; Lehnert *et al*. 2013). An increasing disturbance size, however, homogenizes areas and reduces the forest edge density (Hansson 1994). Future changes in climatic conditions are expected to further intensify disturbance regimes in many ecosystems (Seidl *et al*. 2014; Millar & Stephenson 2015) and will thus exert an important indirect impact of climate change on forest biodiversity. Our study suggests that increasing disturbance frequency and severity (at current disturbance sizes) can compensate negative effects of climate change on selected biodiversity indicators (e.g. tree diversity). This underlines that future studies should take a dynamic and integrative perspective on the potential trajectories of biodiversity beyond correlative relationships with temperature and precipitation. The value of such a perspective is furthermore underlined by finding century‐long lag times in biodiversity responses to climatic drivers (Menendez *et al*. 2006) and in dampening as well as amplifying feedbacks between direct and indirect influences of climate change.

### Implications for conservation management

Our study indicates that local hotspots of biodiversity can shift significantly under climate change, a fact that should be considered more explicitly in future conservation management. Spatio‐temporal analyses as the one presented here can support future conservation planning and foster prospective allocation of resources in conservation management. To increase the robustness of conservation decisions under changing environmental conditions, efforts should focus particularly on areas that are hotspots under both current and changed climatic conditions (see also Rose & Burton 2009). However, it also has to be noted that there are pronounced differences between different hotspot definitions (see Fig. S4), which underline remaining uncertainties in this regard. In the case of the KA‐NP, robust hotspots are located in the central and eastern reaches of the park at low to mid‐elevations in both variants investigated.

Furthermore, addressing a wide range of species groups explicitly is important for conservation management to identify biota particularly at risk from climate change. Based on our analyses, these include Araneae, Carabidae and saproxylic beetles at the KA‐NP. As tree species diversity was also found to decrease, and many phytophages are host‐dependent (Brandle & Brandl 2001), a wide range of indicators might benefit from a coarse filter conservation approach aiming to maintain a diverse forest tree composition. Furthermore, migration corridors as well as temporal connectivity of hotspots on the landscape can help to maintain species threatened by climate change at the regional scale (Fischer, Lindenmayer & Manning 2006). These corridors should be designed particularly to connect current and future hotspots of biodiversity to allow species to relocate in response to changing climatic conditions.

Our findings of positive disturbance impacts on biodiversity underline that intensifying disturbance regimes are congruent with the goals of biodiversity conservation in Central European forests. It is important to note, however, that this positive effect of disturbances can be strongly reduced or even offset by measures such as salvage logging and homogenizing disturbed areas (Lindenmayer & Noss 2006), which is current standard practice in the managed forest ecosystems of Central Europe. For example, the richness of saproxylic beetles as well as wood‐inhabiting fungi has been found to increase after disturbance events, but decreased severely when areas were salvage‐logged (Thorn *et al*. 2014, 2016). Furthermore, such interventions that are usually performed using heavy machinery compact soils and consequently reduce soil fauna and microflora (Marshall 2000). In the light of the importance of biodiversity for the adaptive capacity and response diversity of ecosystems (Mori, Furukawa & Sasaki 2013), a more differentiated perspective on disturbance might be necessary in order to ensure the resilience of forest ecosystems in a rapidly changing world.

## Supporting information


**Fig. S1.** Productivity tests.

**Fig. S2.** Climate sensitivity test.

**Fig. S3.** Potential natural vegetation test.

**Fig. S4.** Additive approach for biodiversity hotspots.

**Table S1.** Coefficient of variation of replicates in each scenario for each biodiversity indicator studied at the end of simulation period.

**Table S2.** Local sensitivity analysis of the empirical species diversity models to changes in climate and tree vegetation.

**Appendix S1.** Initial conditions and drivers add‐on.
