# Supplementary material for: The impacts of climate change and disturbance on spatio‐temporal trajectories of biodiversity in a temperate forest landscape
Source: J Appl Ecol. 2016 Mar 28;54(1):28–38. doi: 10.1111/1365-2664.12644 (PMC5245768; doi:10.1111/1365-2664.12644)
Supplement: Supplementary file 1 — Fig. S1. Productivity tests. [file JPE-54-28-s001.pdf]

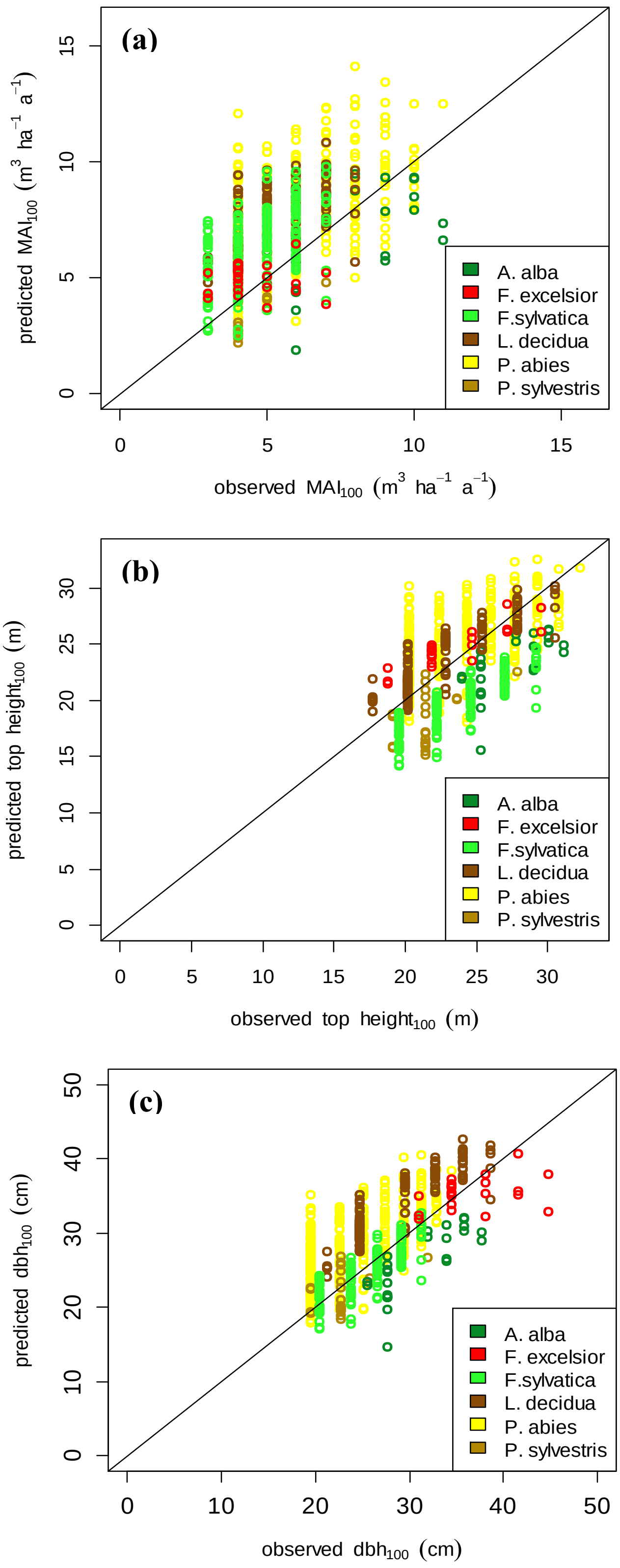

Fig. S1: Productivity comparisons between iLand (predicted) and forest planning data (observed) of single species stands at a forest age of 100 years. Stand-level productivity tests were conducted by sampling 800 ha from the KA-NP landscape, stratified by elevation, and comparing simulated productivity to observations from forest planning data for the six major tree species for which data were available. MAI indicates the mean annual increment and dbh indicates the diameter at breast height.
