# Supplementary material for: The impacts of climate change and disturbance on spatio‐temporal trajectories of biodiversity in a temperate forest landscape
Source: J Appl Ecol. 2016 Mar 28;54(1):28–38. doi: 10.1111/1365-2664.12644 (PMC5245768; doi:10.1111/1365-2664.12644)
Supplement: Supplementary file 2 — Fig. S2. Climate sensitivity test. [file JPE-54-28-s002.pdf]

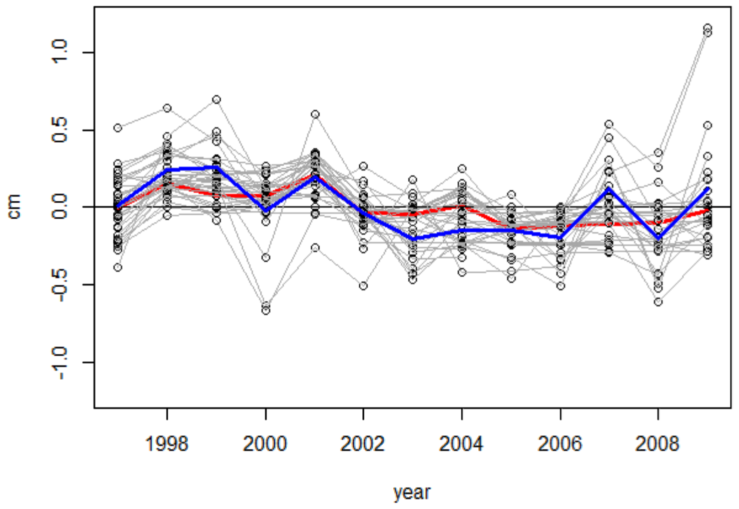

Fig. S2: Model sensitivity to interannual climate variation. To test the model's ability to correctly capture the effect of interannual climate variation at the level of individual trees, we used data from an intensive forest monitoring plot located within the Kalkalpen Nationalpark, for which the dbh of 15 trees (grey) has been monitored using automated dendrometers over a 14 year period The figure presents a moving average over growth anomalies with a time lag of one year. red: iLand simulation, blue: observed by using automated dendrometers. Gleichläufigkeit=0.690.
