# Supplementary material for: The impacts of climate change and disturbance on spatio‐temporal trajectories of biodiversity in a temperate forest landscape
Source: J Appl Ecol. 2016 Mar 28;54(1):28–38. doi: 10.1111/1365-2664.12644 (PMC5245768; doi:10.1111/1365-2664.12644)
Supplement: Supplementary file 3 — Fig. S3. Potential natural vegetation test. [file JPE-54-28-s003.pdf]

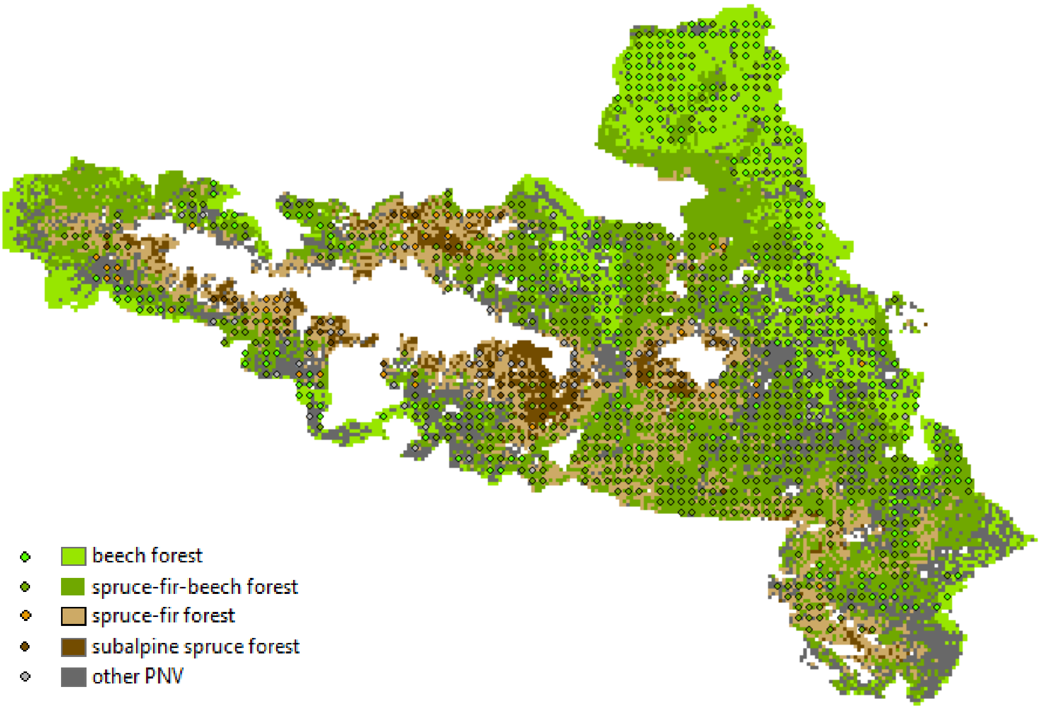

Fig. S3: Comparison between simulated potential natural vegetation (PNV, continuous map) and expectations from inventory plots (circles). For this evaluation exercise we simulated the entire landscape for 2,500 years starting from bare ground under baseline climate conditions, and compared the simulated equilibrium vegetation composition with expectations for the PNV recorded at 1,110 inventory plots.
