# Supplementary material for: The impacts of climate change and disturbance on spatio‐temporal trajectories of biodiversity in a temperate forest landscape
Source: J Appl Ecol. 2016 Mar 28;54(1):28–38. doi: 10.1111/1365-2664.12644 (PMC5245768; doi:10.1111/1365-2664.12644)
Supplement: Supplementary file 4 — Fig. S4. Additive approach for biodiversity hotspots. [file JPE-54-28-s004.pdf]

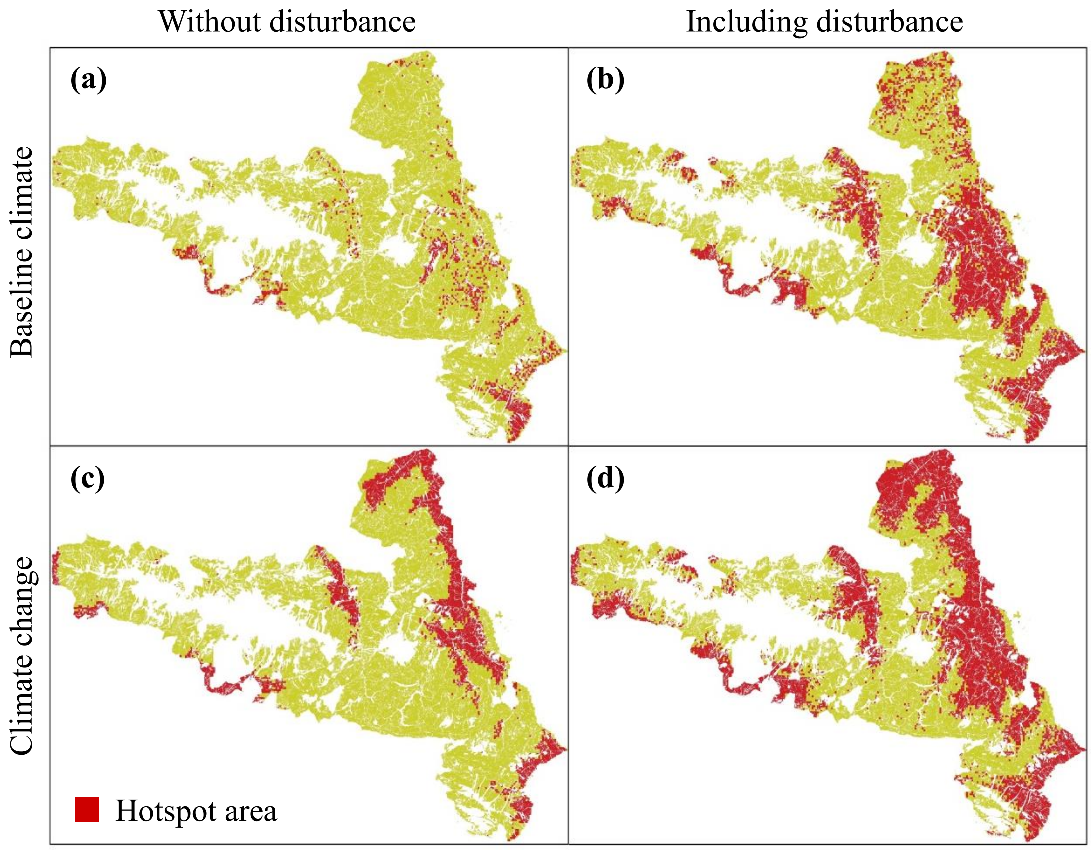

Fig. S4: Additive biodiversity hotspots of the forested area of the Kalkalpen National Park after 1000 years simulation. As an alternative to the threshold-based hotspot definition presented in the main text, we here aggregated percentiles and averaged the sum over the eleven diversity indicators to derive a diversity scalar [0,1]. In this additive approach, we defined biodiversity hotspots as areas exceeding a value of 0.6. By summing up the percentiles, this approach is less sensitive to changes of each individual indicator, but allows trade-offs between high and low local diversity. In contrast to the threshold-based hotspot definition, the additive definition indicated a more distinctive spatial overlap in hotspot areas under current and future climate (i.e. 66.3% and 27.9% of hotspots areas under changed climatic conditions overlap with hotspot areas under baseline climate conditions when disturbance is in- or excluded, respectively). In the baseline climate scenario the hotspot areas were mostly beech dominated, but changed to predominantly oak dominated areas under a changing climate. In contrast to the fixed threshold hotspot definition, here climate change even increased the area defined as hotspot significantly ($p<0.001$). Disturbance was again found to increase biodiversity hotspots ($p<0.001$) with disturbance-induced increases in area defined as hotspot by 266.9% and 128.5%, respectively, under baseline and changed climatic conditions.
