# Supplementary material for: The impacts of climate change and disturbance on spatio‐temporal trajectories of biodiversity in a temperate forest landscape
Source: J Appl Ecol. 2016 Mar 28;54(1):28–38. doi: 10.1111/1365-2664.12644 (PMC5245768; doi:10.1111/1365-2664.12644)
Supplement: Supplementary file 7 — Appendix S1. Initial conditions and drivers add‐on. [file JPE-54-28-s007.docx]

INITIAL CONDITIONS AND DRIVERS ADD-ON

Soil

Soil data were derived from inventory plots distributed over the national park on a regular 300 m × 300 m grid. 710 and 688 inventory plots were used to develop statistical models for soil depth (Fig. 1) and soil type (Fig. 2), using linear and logistic regression respectively (Kobler 2004). These relationships were subsequently used to derive wall-to-wall estimate of these parameters for the landscape. Surveys on soil texture (N=622) were aggregated by soil type and resampled for the whole landscape. Plant available nitrogen values were derived from a dataset previously developed for the grid of the Austrian National Forest Inventory (Seidl, Rammer & Lexer 2009), and were imputed to the study area by a stratified sampling over ecoregion, elevation, soil depth, aspect and slope (N=557).


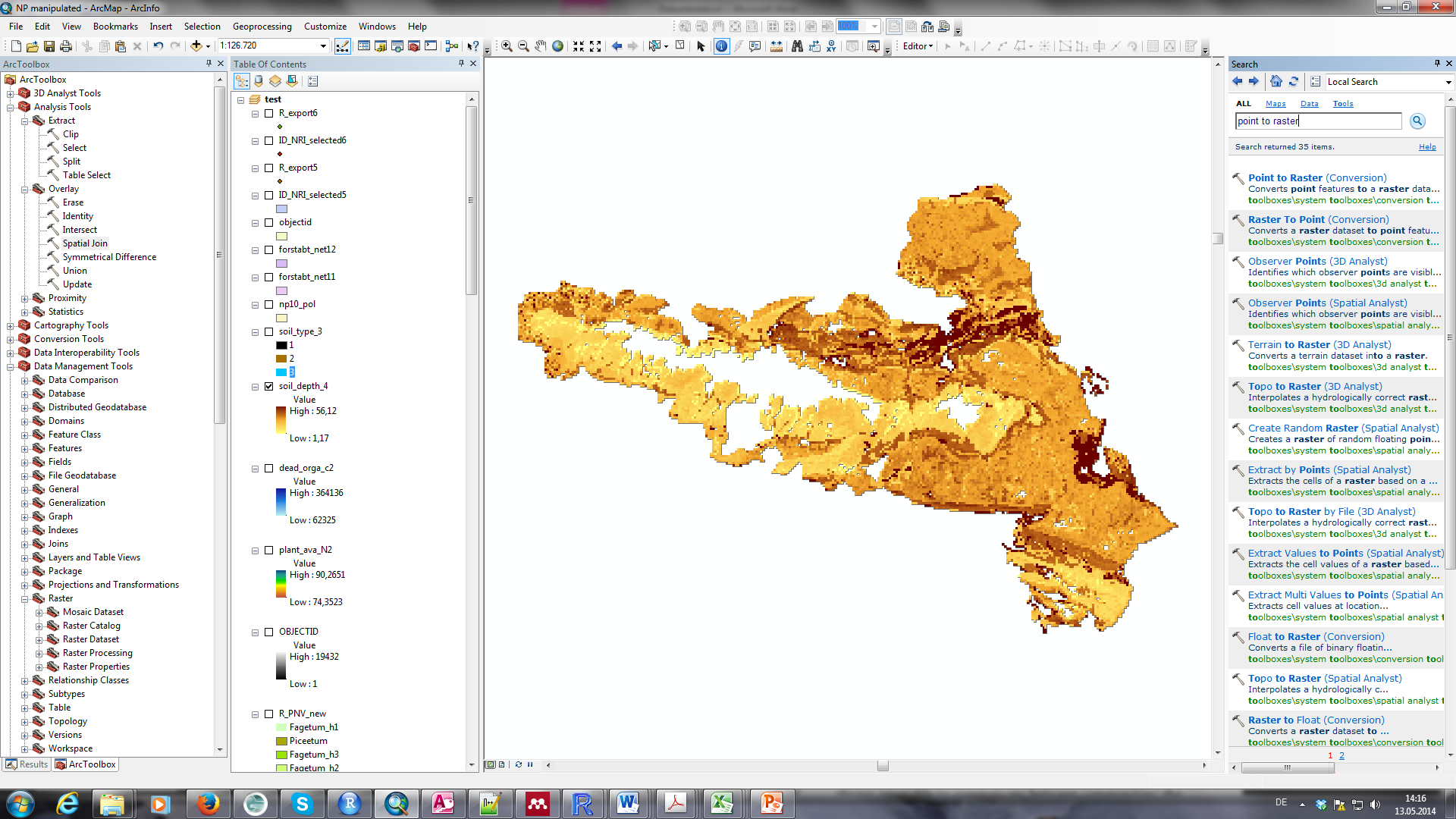

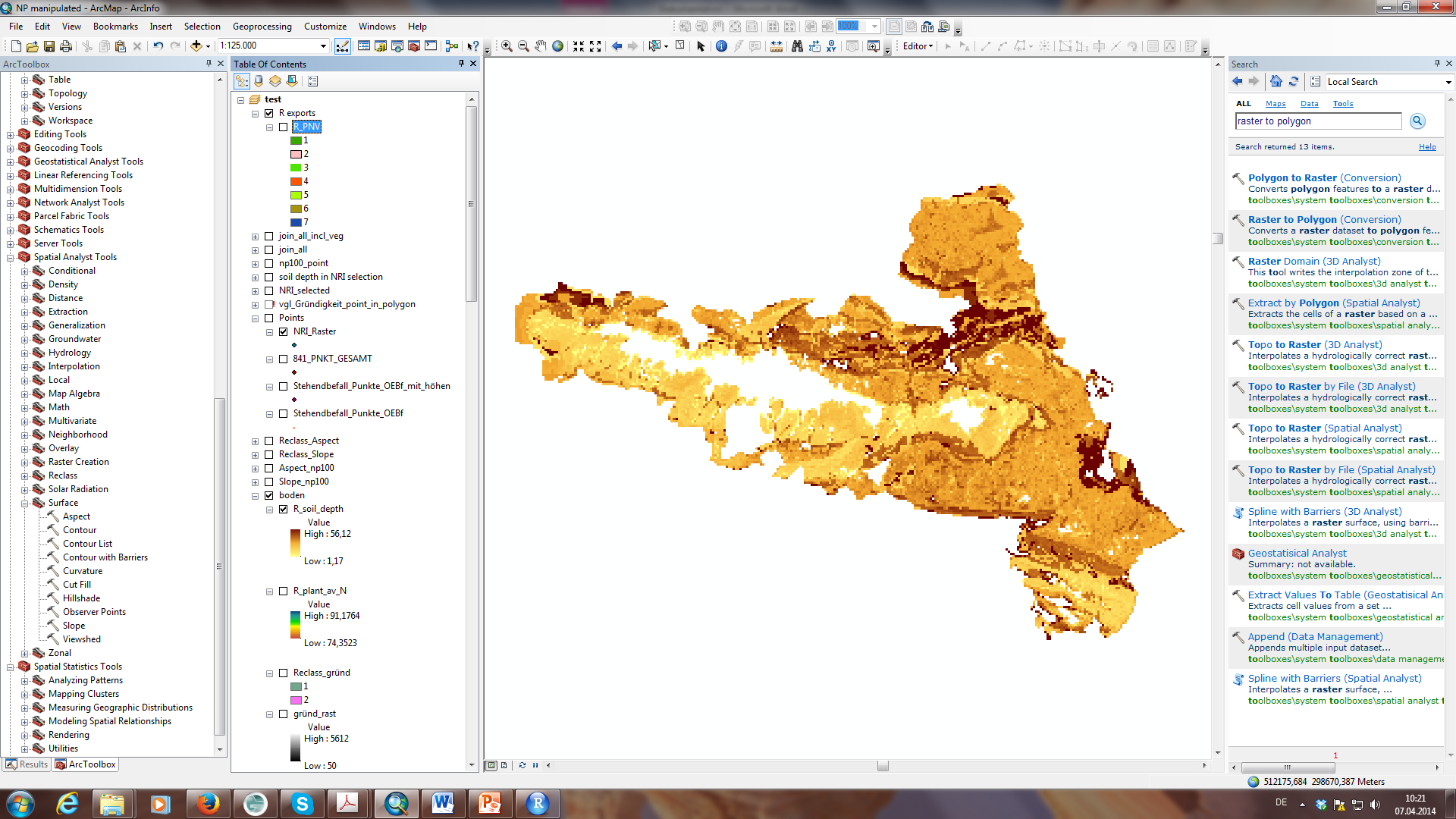


56.12 cm

1.17 cm

Fig. A1. Effective soil depth according to Kobler (2004).


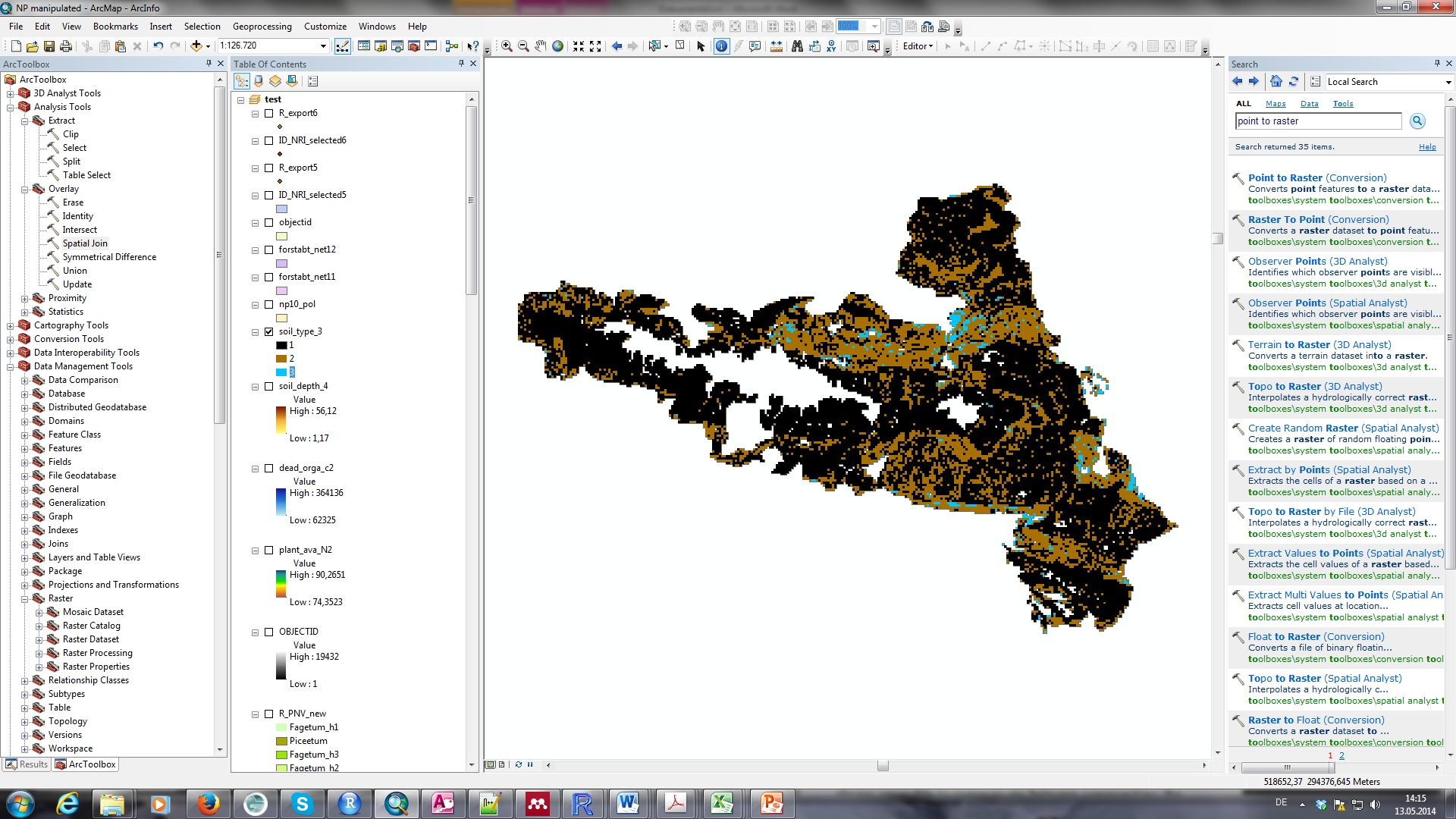

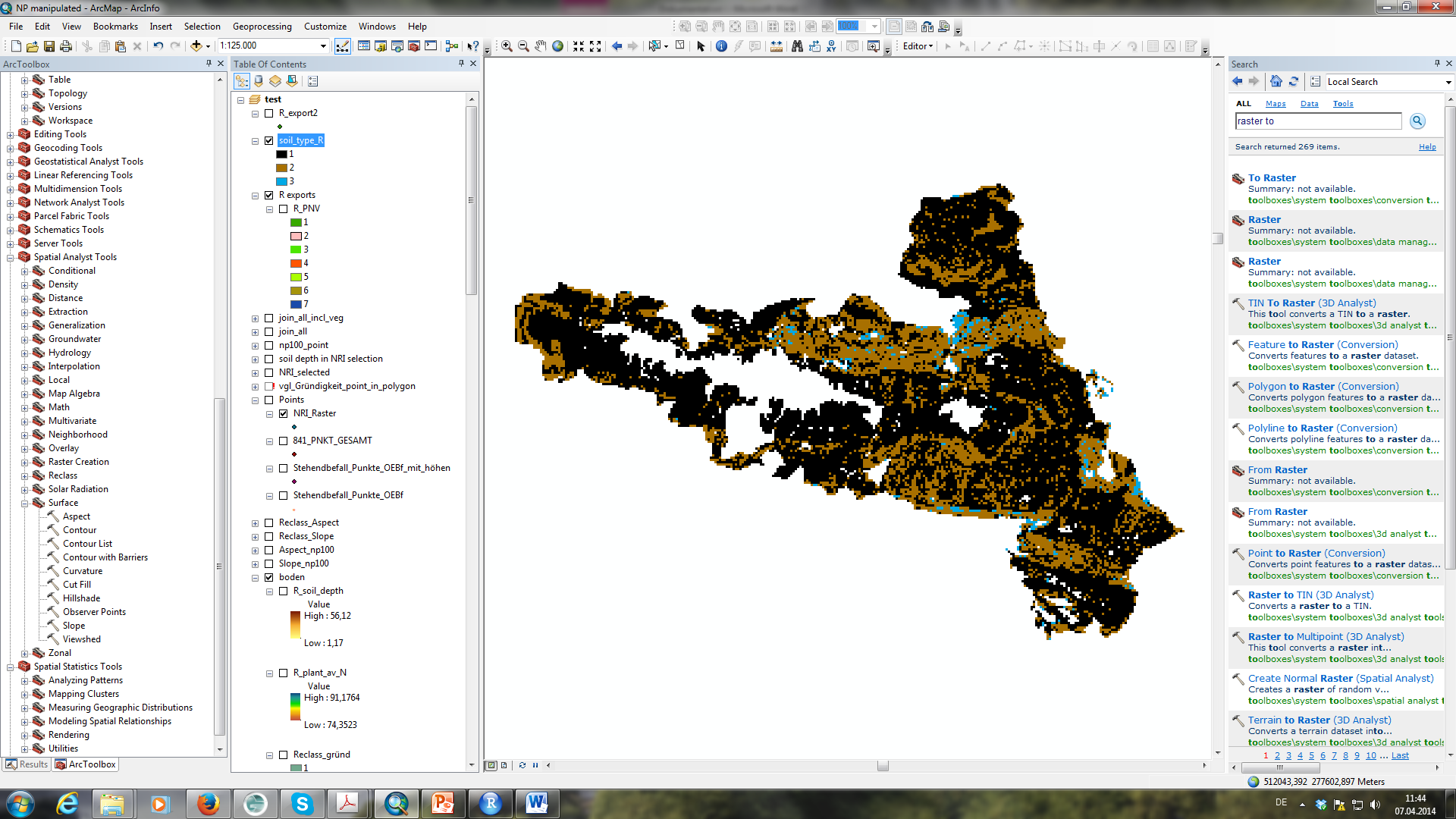


Rendzic Leptosols Leptosols

Cromic Cambisols

Gley types

Fig. A2. Soil types according to Kobler (2004).

Climate

The years 1950 – 2010 were selected as the baseline for the analysis of climate change effects (reference period, see examples for climate input parameters in Fig. 4 and Fig. 5). Three regionally downscaled climate change scenarios, representing different combinations of global and regional circulation models under A1B forcing, were studied: CNRM-RM4.5 (Radu, Déqué & Somot 2008) driven by the global climate models (GCM) ARPEGE and MPI-REMO (Jacob 2001) as well as ICTP-RegCM3 (Pal *et al.* 2007) driven by the GCM ECHAM5. Climate changed transiently in these scenarios until the end of the 21^st^ century (temperature change of between 3.1°C and 3.3°C, and precipitation change of between –89 mm and + 141 mm in 2080 – 2099 relative to the baseline period). A stabilization of climate conditions at the level of 2080 – 2099 was assumed for the years beyond 2100.


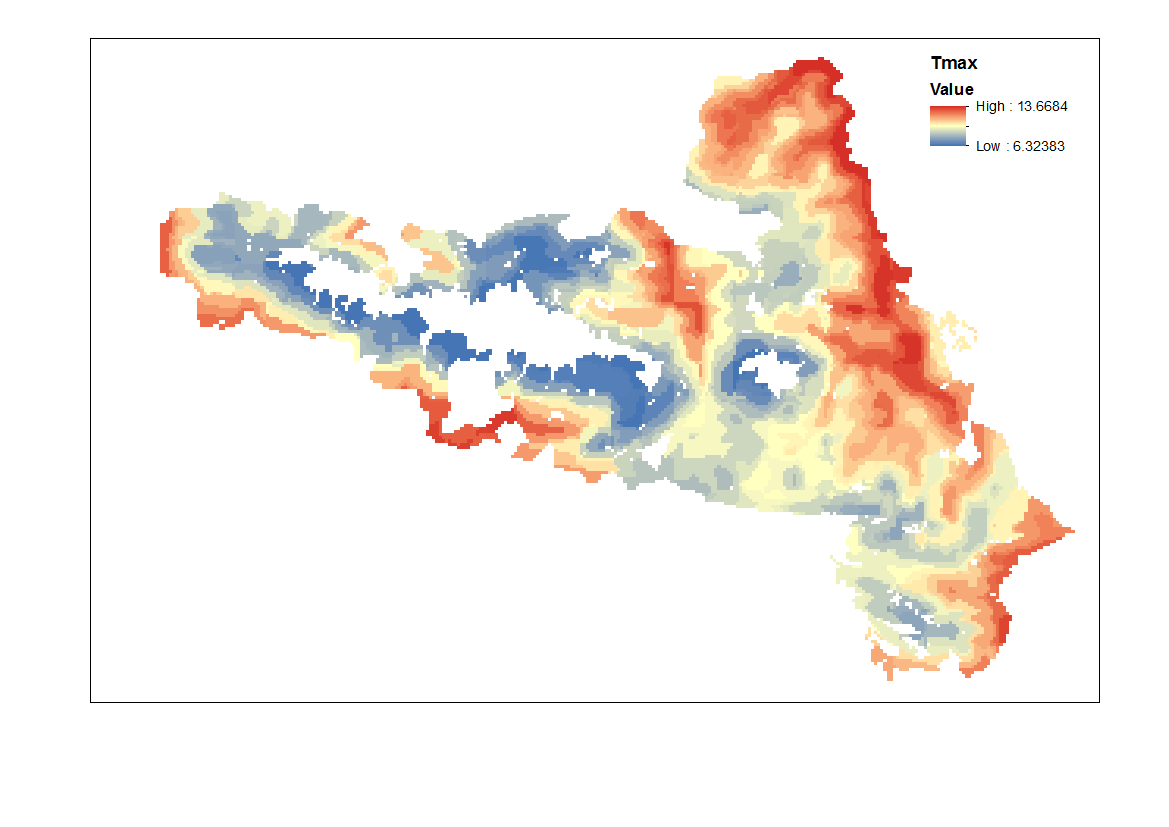

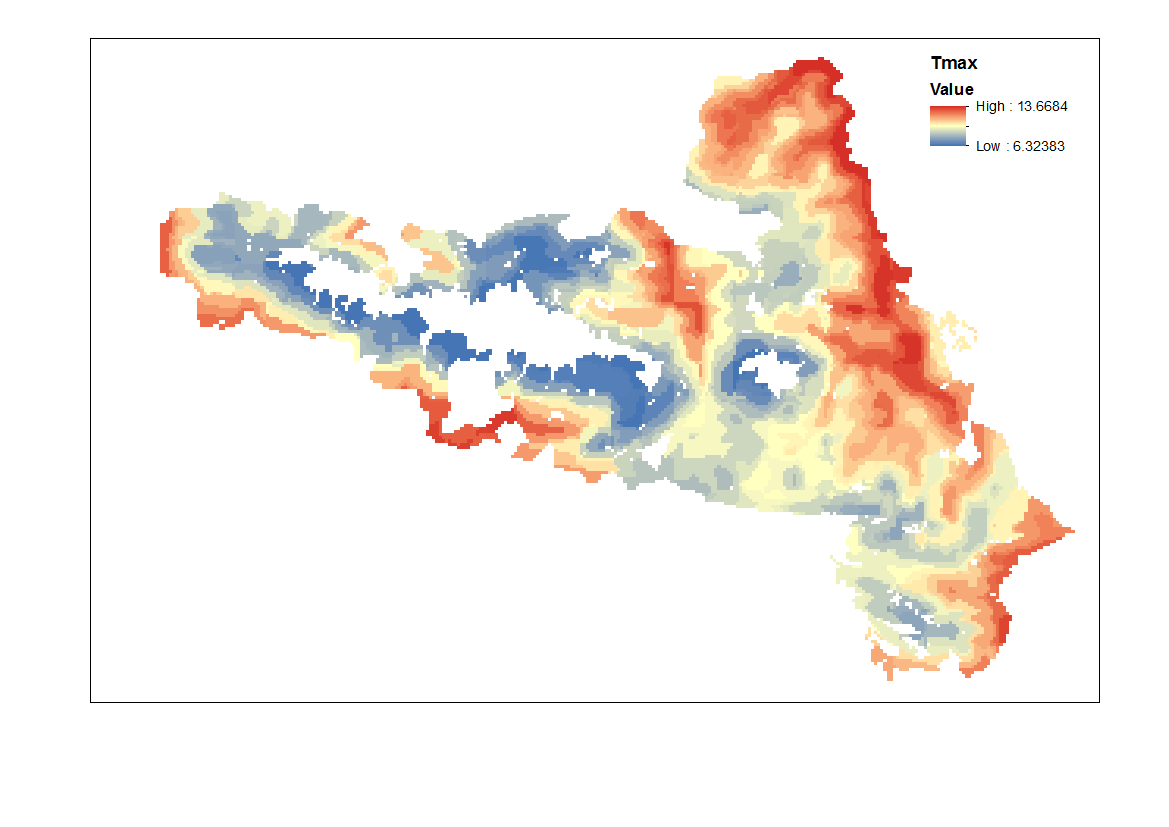


13.7 °C

6.5 °C

Fig. A3. Mean maximum temperature under baseline climate conditions.


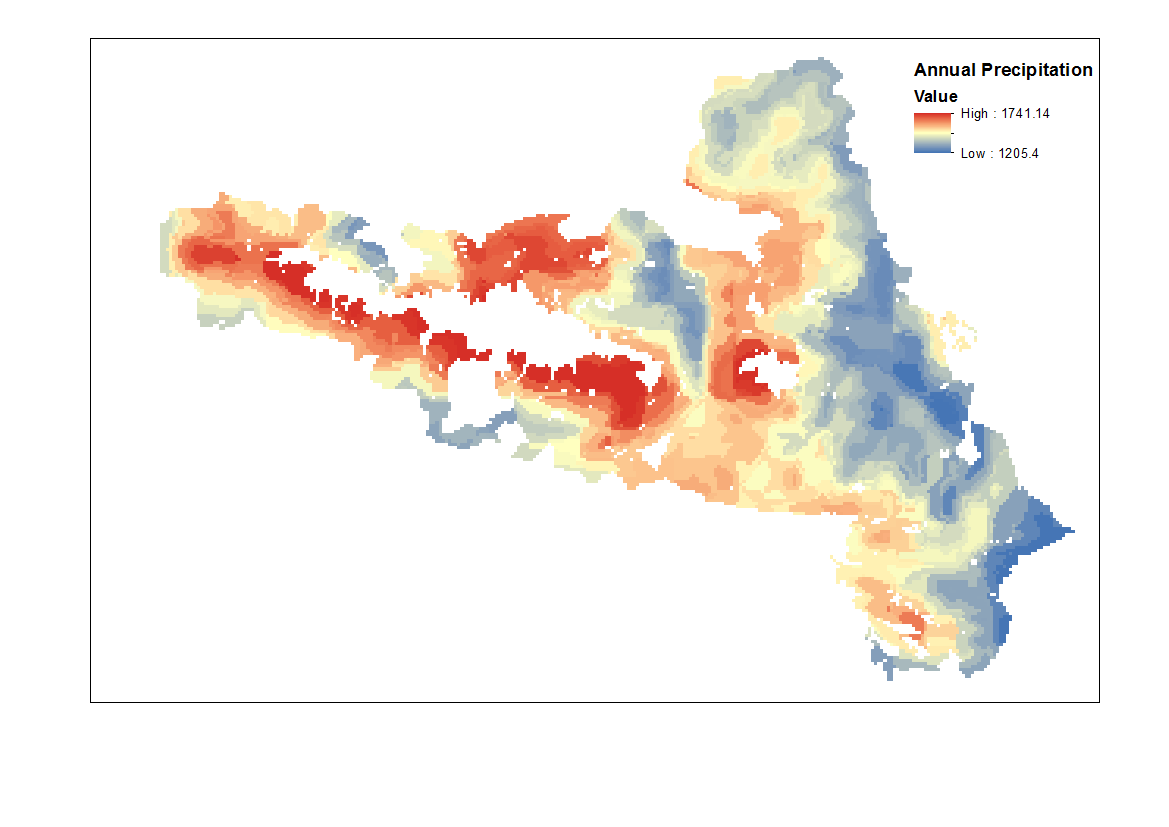

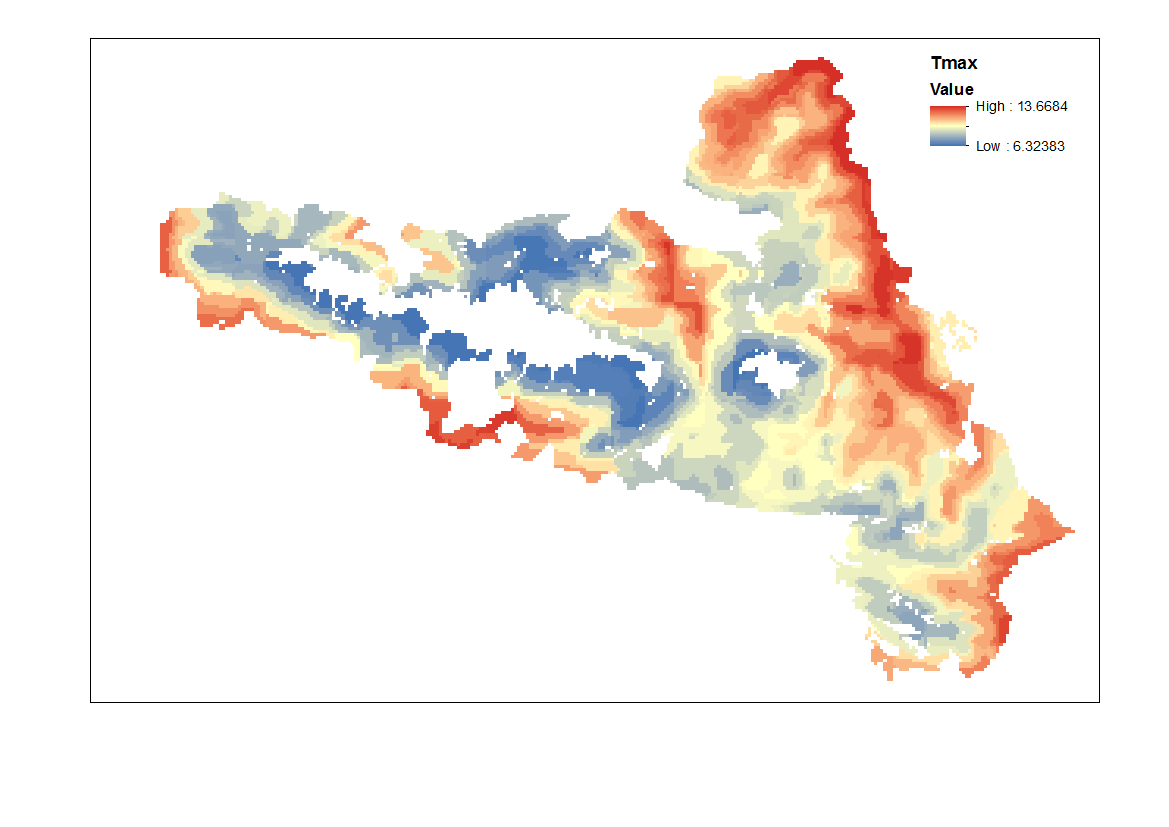


1702 mm

1178 mm

Fig. A4. Annual precipitation sum under baseline climate conditions.

Vegetation

Information on current vegetation was derived via integrating various data sources. First, we defined stand polygons (median stand size: 1.4 ha) based on aerial photo analysis with rough estimates for tree species composition. Then we used terrestrial inventory data (N=1,122) to refine tree species composition as well as to derive diameter at breast height (dbh) and the number of trees per ha within each stand polygons. To establish stand age we used information from forest inventory and planning data. Airborne LiDAR (light detection and ranging) was improved by forest inventory data to estimate tree heights of forest stands (Fig. 6). LiDAR and forest inventories were further used to derive the stockable area within a stand (i.e. based on these data sources, we excluded areas on a 10 m × 10 m where trees cannot grow, for example due to rocks on these areas). LiDAR data also served to determine the position of trees for initialization of the simulation model (i.e. we did not assume a uniform distribution of trees over the stands, but accounted for gaps and lumps of trees within the stands). Altogether, we initialized more than 2 10^6^ trees from 17 different species on an area of 13.865 ha. All these data sources pertained to the end of the 1990s or early 2000s, and were combined to determine the state of tree vegetation at KA-NP in the year 1999.


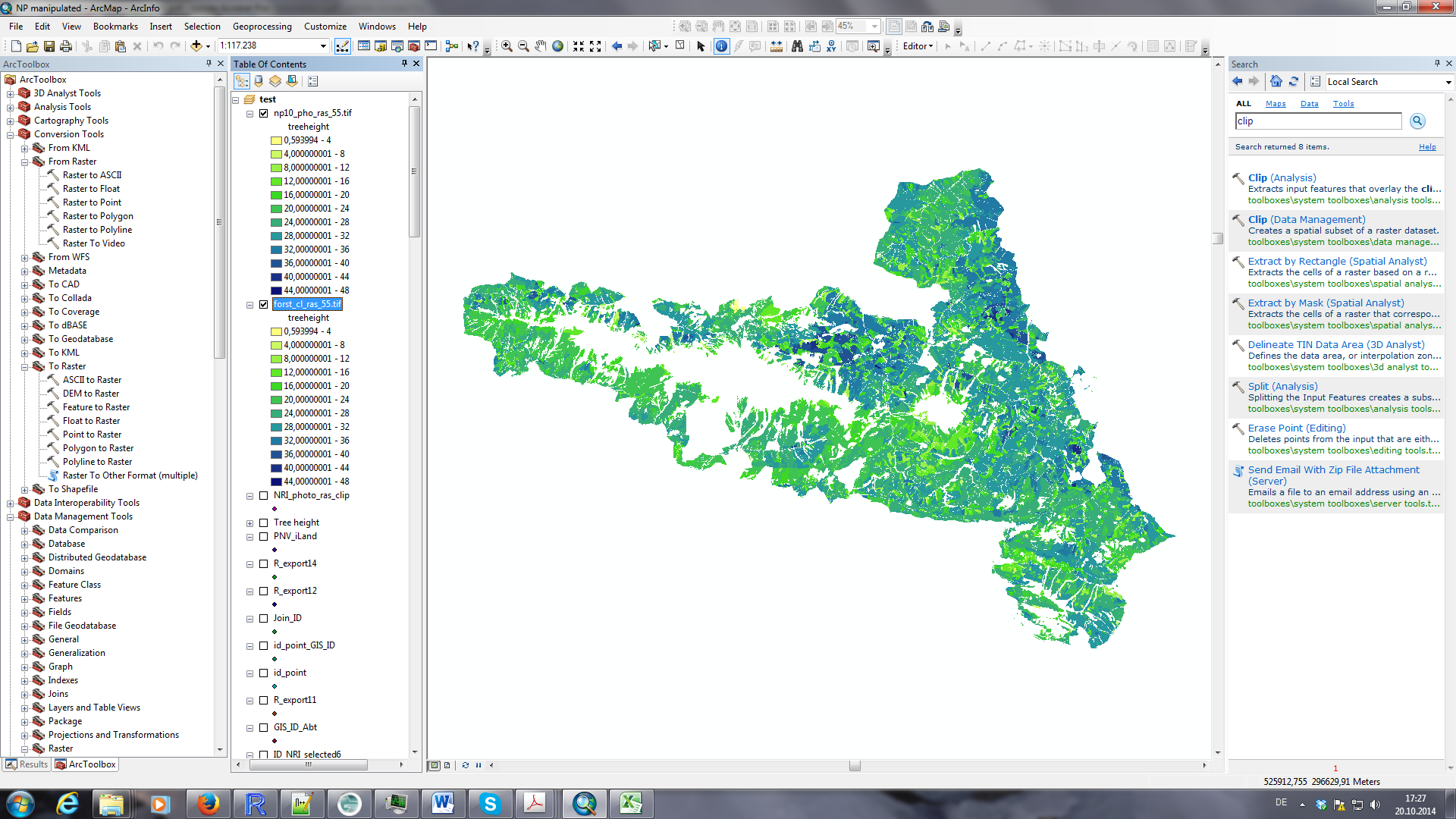

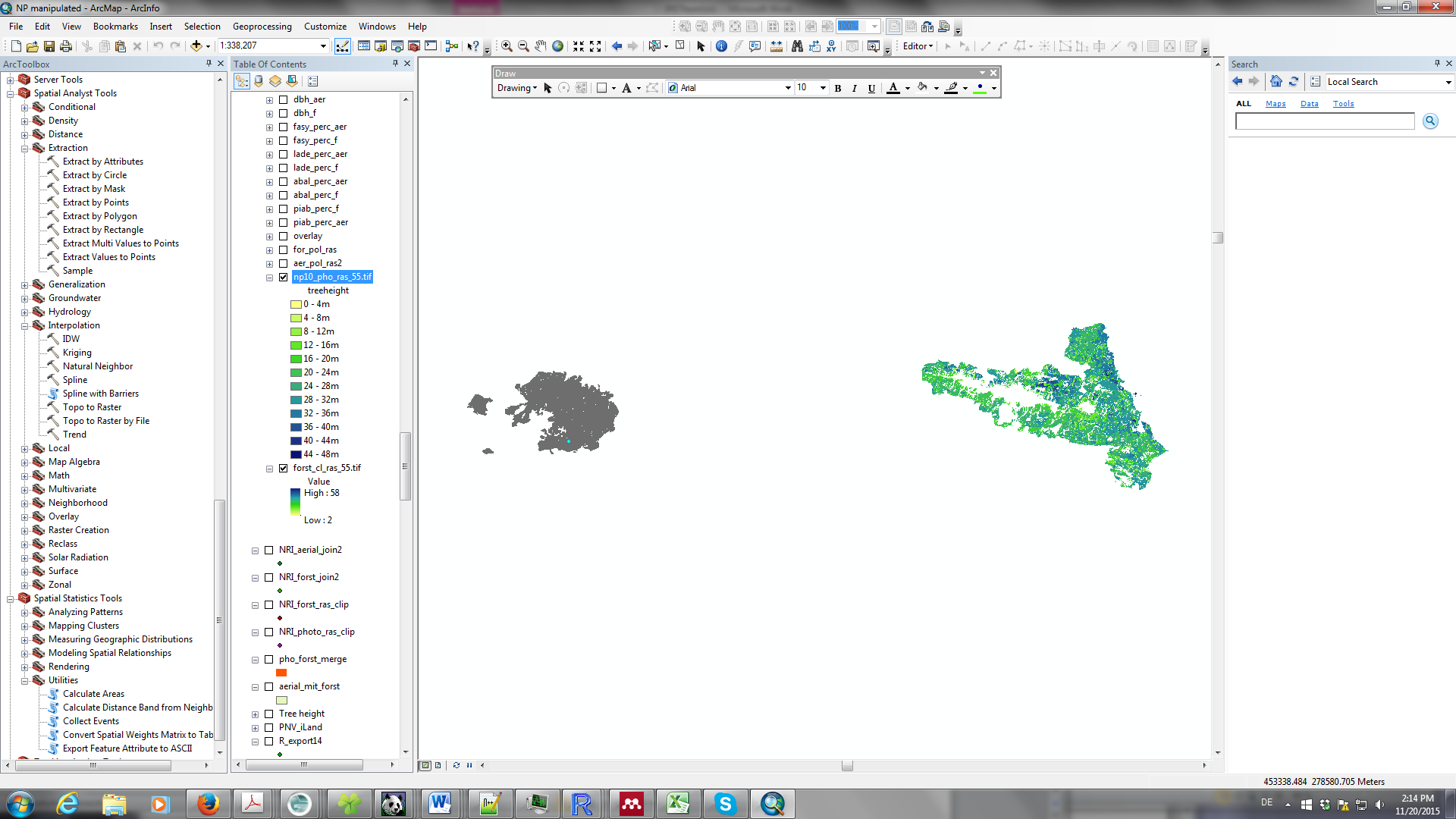


44.6 m

0.6 m

Fig. A5. Dominant tree height within stand polygons.

**References**

Jacob, D. (2001) A note to the simulation of the annual and inter-annual variability of the water budget over the Baltic Sea drainage basin. *Meteorology and Atmospheric Physics*, **77**, 61–73.

Kobler, J (2004) Risikokarten als Planungsgrundlage für Flächenbewirtschaftung und Tourismuslenkung im Nationalpark Kalkalpen Oberösterreich. [Faculty of Earth Sciences, Geography and Astronomy](https://fgga.univie.ac.at/en/home-neu/), University of Vienna. 1-304.

Pal, J.S., Giorgi, F., Bi, X., Elguindi, N., Solmon, F., Rauscher, S.A., Gao, X., Francisco, R., Zakey, A., Winter, J., Ashfaq, M., Syed, F.S., Sloan, L.C., Bell, J.L., Diffenbaugh, N.S., Karmacharya, J., Konaré, A., Martinez, D., da Rocha, R.P. & Steiner, A.L. (2007) Regional climate modeling for the developing world: The ICTP RegCM3 and RegCNET. *Bulletin of the American Meteorological Society*, **88**, 1395–1409.

Radu, R., Déqué, M. & Somot, S. (2008) Spectral nudging in a spectral regional climate model. *Tellus A*, **60**, 898–910.

Seidl, R., Rammer, W. & Lexer, M.J. (2009) Schätzung von Bodenmerkmalen und Modellparametern für die Waldökosystemsimulation auf Basis einer Großrauminventur. *Allgemeine Forst- und Jagdzeitung*, **180**, 35–44.
